# Supplementary material for: Immunohistological detection of small particles of Echinococcus multilocularis and Echinococcus granulosus in lymph nodes is associated with enlarged lymph nodes in alveolar and cystic echinococcosis
Source: PLoS Negl Trop Dis. 2020 Dec 28;14(12):e0008921. doi: 10.1371/journal.pntd.0008921 (PMC7769273; doi:10.1371/journal.pntd.0008921)
Supplement: S1 Table — (DOCX) [file pntd.0008921.s001.docx]

**S1 Table. Negative controls for mAb EmG3**

| **tissue material** | | **mAb EmG3 immunohistochemistry** |
| --- | --- | --- |
| tumor necrosis | colon cancer (n=1) | negative |
|  | adenocarcinoma (gastroesophageal junction) (n=1) | negative |
|  | bronchial carcinoma (n=2)) | negative |
|  | rectal cancer (n=1) | negative |
|  | Klatskin tumor (n=1) | weak staining of liver tissue |
| necrosis in tuberculosis (n=3) | | negative |
| rheumatoid nodule with necrotic area (n=2) | | negative |
| lymphadenitis with necrotic area(n=1) | | negative |
| immunoreactive lymph node(n=1) | | negative |
| tonsil (n=4) | | negative |
| gallbladder (n=1) | | weak stain of epithelia |
| liver infraction with necrotic area (n=1) | | weak staining |
| lymph node by sarcoidosis(n=2) | | negative |
| parasites (*Trichuris suis, Taenia solium, Strongyloides stercoralis, Enterobius vermicularis*; n=4) | | negative, except weak staining of *Trichuris suis* |
