# Supplementary material for: Immunohistological detection of small particles of Echinococcus multilocularis and Echinococcus granulosus in lymph nodes is associated with enlarged lymph nodes in alveolar and cystic echinococcosis
Source: PLoS Negl Trop Dis. 2020 Dec 28;14(12):e0008921. doi: 10.1371/journal.pntd.0008921 (PMC7769273; doi:10.1371/journal.pntd.0008921)
Supplement: S2 Table — (DOCX) [file pntd.0008921.s002.docx]

**S2 Table. IgM-isotype controls for mAb EmG3**

| **tissue material** | **IgM-isotype immunohistochemistry** |
| --- | --- |
| adenocarcinoma of the lung (n=1) | negative |
| muscle (n=1) | negative |
| liver (n=2) | staining of lipofuscin |
| lymph node (n=1) | negative |
| *Trichuris suis* (n=1) | weak staining |
| AE lesions (n=3) | negative |
| CE lesions (n=7) | negative |
