# Supplementary material for: Immunohistological detection of small particles of Echinococcus multilocularis and Echinococcus granulosus in lymph nodes is associated with enlarged lymph nodes in alveolar and cystic echinococcosis
Source: PLoS Negl Trop Dis. 2020 Dec 28;14(12):e0008921. doi: 10.1371/journal.pntd.0008921 (PMC7769273; doi:10.1371/journal.pntd.0008921)
Supplement: S3 Table — (DOCX) [file pntd.0008921.s003.docx]

**S3 Table. Data of lymph nodes (n=95) of 25 patients with alveolar echinococcosis**

| **lymph node** | **mAb EmG3** | **germinal center** | **sinus** | **area (mm²)** | **localization** | **serology** | **PNM** |
| --- | --- | --- | --- | --- | --- | --- | --- |
| **lymph node 1**  **(patient 101; slide 1)** | positive | positive | positive | 276 | pancreas | negative | P2N0M0 |
| **2 (102; 1)** | positive | positive | negative | 45 | liver | positive | N/A |
| **3 (102; 1)** | negative | negative | negative | 80 | liver | positive | N/A |
| **4 (102; 2)** | positive | positive | negative | 56 | gall bladder | positive | N/A |
| **5 (102; 2)** | positive | positive | negative | 82.5 | gall bladder | positive | N/A |
| **6 (102; 2)** | positive | positive | negative | 4 | gall bladder | positive | N/A |
| **7 (103; 1)** | positive | positive | negative | 14 | gall bladder | negative | N/A |
| **8 (103; 1)** | positive | positive | positive | 26.25 | gall bladder | negative | N/A |
| **9 (104; 1)** | positive | positive | positive | 150 | gall bladder | positive | P2N0M0 |
| **10 (104; 1)** | positive | positive | positive | 12 | gall bladder | positive | P2N0M0 |
| **11 (104; 1)** | positive | positive | positive | 7 | gall bladder | positive | P2N0M0 |
| **12 (105; 1)** | positive | positive | positive | 140 | gall bladder | positive | N/A |
| **13 (105; 1)** | positive | positive | positive | 50 | gall bladder | positive | N/A |
| **14 (105; 1)** | positive | positive | positive | 210 | gall bladder | positive | N/A |
| **15 (106; 1)** | positive | positive | positive | 160 | liver | positive | N/A |
| **16 (106; 1)** | positive | positive | negative | 40 | liver | positive | N/A |
| **17 (106; 1)** | negative | negative | negative | 128 | liver | positive | N/A |
| **18 (107; 1)** | positive | positive | positive | 84 | liver | positive | P3N0M0 |
| **19 (107; 1)** | positive | negative | positive | 4 | liver | positive | P3N0M0 |
| **20 (107; 1)** | negative | negative | negative | 2 | liver | positive | P3N0M0 |
| **21 (107; 2)** | positive | positive | positive | 84 | liver | positive | P3N0M0 |
| **22 (107; 2)** | negative | negative | negative | 1 | liver | positive | P3N0M0 |
| **23 (108; 1)** | negative | negative | negative | 72 | liver | positive | P3N0M0 |
| **24 (108; 1)** | negative | negative | negative | 208 | liver | positive | P3N0M0 |
| **25 (108; 2)** | positive | positive | negative | 91 | liver | positive | P3N0M0 |
| **26 (108; 2)** | negative | negative | negative | 14 | liver | positive | P3N0M0 |
| **27 (108; 2)** | negative | negative | negative | 18 | liver | positive | P3N0M0 |
| **28 (108; 2)** | negative | negative | negative | 15 | liver | positive | P3N0M0 |
| **29 (108; 2)** | negative | negative | negative | 38.5 | liver | positive | P3N0M0 |
| **30 (108; 2)** | negative | negative | negative | 36 | liver | positive | P3N0M0 |
| **31 (108; 2)** | negative | negative | negative | 4 | liver | positive | P3N0M0 |
| **32 (109; 1)** | positive | positive | positive | 168 | liver | negative | P2N0M0 |
| **33 (109; 1)** | negative | negative | negative | 65 | liver | negative | P2N0M0 |
| **34 (109; 1)** | negative | negative | negative | 15 | liver | negative | P2N0M0 |
| **35 (109; 2)** | positive | positive | positive | 24.5 | liver | negative | P2N0M0 |
| **36 (109; 2)** | positive | positive | positive | 88 | liver | negative | P2N0M0 |
| **37 (109; 2)** | positive | positive | positive | 200 | liver | negative | P2N0M0 |
| **38 (109; 3)** | negative | negative | negative | 12 | liver | negative | P2N0M0 |
| **39 (109; 3)** | negative | negative | negative | 50 | liver | negative | P2N0M0 |
| **40 (109; 3)** | negative | negative | negative | 12 | liver | negative | P2N0M0 |
| **41 (109; 3)** | negative | negative | negative | 12 | liver | negative | P2N0M0 |
| **42 (110; 1)** | positive | positive | positive | 40 | liver | positive | p2N0M0 |
| **43 (111; 1)** | negative | negative | negative | 28 | gall bladder | positive | N/A |
| **44 (111; 2)** | negative | negative | negative | 49 | diaphragm | positive | N/A |
| **45 (111; 2)** | negative | negative | negative | 6 | diaphragm | positive | N/A |
| **46 (112; 1)** | negative | negative | negative | 18 | gall bladder | positive | P2N0M0 |
| **47 (113; 1)** | positive | positive | negative | 24 | gall bladder | positive | P3N1M0 |
| **48 (113; 1)** | positive | positive | negative | 64 | gall bladder | positive | P3N1M0 |
| **49 (114; 1)** | positive | positive | negative | 3 | vena cava | positive | P2N0M0 |
| **50 (114; 1)** | positive | positive | negative | 3 | vena cava | positive | P2N0M0 |
| **51 (114; 2)** | positive | positive | negative | 12 | gall bladder | positive | P2N0M0 |
| **52 (114; 3)** | positive | positive | negative | 45 | liver | positive | P2N0M0 |
| **53 (115; 1)** | positive | positive | positive | 32.5 | stomach | positive | P2N0M0 |
| **54 (115; 1)** | positive | positive | positive | 6.25 | stomach | positive | P2N0M0 |
| **55 (115; 1)** | negative | negative | negative | 12 | stomach | positive | P2N0M0 |
| **56 (115; 2)** | positive | positive | negative | 35 | stomach | positive | P2N0M0 |
| **57 (115; 2)** | negative | negative | negative | 4.5 | stomach | positive | P2N0M0 |
| **58 (116; 1)** | positive | positive | positive | 15 | liver | positive | P4NxMx |
| **59 (116; 1)** | positive | positive | positive | 88 | liver | positive | P4NxMx |
| **60 (116; 1)** | positive | positive | negative | 160 | liver | positive | P4NxMx |
| **61 (116; 1)** | positive | positive | positive | 52 | liver | positive | P4NxMx |
| **62 (116; 2)** | positive | positive | negative | 88 | liver | positive | P4NxMx |
| **63 (116; 2)** | positive | positive | negative | 4 | liver | positive | P4NxMx |
| **64 (116; 3)** | positive | positive | negative | 16 | liver | positive | P4NxMx |
| **65 (116; 3)** | positive | positive | negative | 56 | liver | positive | P4NxMx |
| **66 (116; 3)** | positive | positive | negative | 35 | liver | positive | P4NxMx |
| **67 (116; 4)** | positive | positive | positive | 32 | liver | positive | P4NxMx |
| **68 (116; 4)** | positive | positive | positive | 63 | liver | positive | P4NxMx |
| **69 (116; 4)** | positive | positive | negative | 12 | liver | positive | P4NxMx |
| **70 (116; 4)** | positive | positive | positive | 50 | liver | positive | P4NxMx |
| **71 (117; 1)** | positive | positive | negative | 27 | liver | positive | P2N1M0 |
| **72 (117; 2)** | positive | positive | positive | 84 | liver | positive | P2N1M0 |
| **73 (118; 1)** | negative | negative | negative | 12 | liver | positive | P2N0M0 |
| **74 (118; 2)** | negative | negative | negative | 165 | gall bladder | positive | P2N0M0 |
| **75 (118; 2)** | negative | negative | negative | 91 | gall bladder | positive | P2N0M0 |
| **76 (119; 1)** | positive | positive | negative | 1 | liver | positive | N/A |
| **77 (119; 2)** | positive | positive | positive | 40 | liver | positive | N/A |
| **78 (120; 1)** | positive | positive | positive | 240 | liver | positive | P2N0M0 |
| **79 (120; 2)** | positive | positive | positive | 238 | liver | positive | P2N0M0 |
| **80 (120; 3)** | positive | positive | positive | 150 | liver | positive | P2N0M0 |
| **81 (120; 3)** | positive | positive | negative | 20 | liver | positive | P2N0M0 |
| **82 (121; 1)** | positive | positive | negative | 360 | liver | positive | P4N0M0 |
| **83 (121; 2)** | negative | negative | negative | 30 | liver | positive | P4N0M0 |
| **84 (121; 2)** | positive | positive | negative | 33 | liver | positive | P4N0M0 |
| **85 (121; 2)** | negative | negative | negative | 35 | liver | positive | P4N0M0 |
| **86 (121; 2)** | negative | negative | negative | 8 | liver | positive | P4N0M0 |
| **87 (122; 1)** | negative | negative | negative | 36 | vena cava | positive | P2N0M0 |
| **88 (123; 1)** | positive | positive | positive | 80 | gall bladder | positive | P4N1M0 |
| **89 (123; 1)** | positive | positive | positive | 24 | gall bladder | positive | P4N1M0 |
| **90 (123; 2)** | positive | positive | negative | 84 | liver | positive | P4N1M0 |
| **91 (123; 3)** | positive | positive | positive | 378 | liver | positive | P4N1M0 |
| **92 (123; 4)** | positive | positive | positive | 360 | liver | positive | P4N1M0 |
| **93 (124; 1)** | positive | positive | positive | 15 | gall bladder | positive | P4N1M0 |
| **94 (125; 1)** | negative | negative | negative | 1.5 | gall bladder | positive | P2N1M1 |
| **95 (125; 2)** | positive | positive | positive | 64 | spleen | positive | P2N1M1 |

*N/A: not available*
