# Supplementary material for: Immunohistological detection of small particles of Echinococcus multilocularis and Echinococcus granulosus in lymph nodes is associated with enlarged lymph nodes in alveolar and cystic echinococcosis
Source: PLoS Negl Trop Dis. 2020 Dec 28;14(12):e0008921. doi: 10.1371/journal.pntd.0008921 (PMC7769273; doi:10.1371/journal.pntd.0008921)
Supplement: S4 Table — (DOCX) [file pntd.0008921.s004.docx]

**S4 Table. Data of lymph nodes (n=41) of 12 patients with cystic echinococcosis**

| **lymph node** | **mAb EmG3** | **germinal center** | **sinus** | **area (mm²)** | **localization** | **serology** |
| --- | --- | --- | --- | --- | --- | --- |
| **lymph node 1  (patient 201; slide 1)** | positive | positive | positive | 4.5 | lung | N/A |
| **2 (201; 1)** | positive | positive | positive | 12 | lung | N/A |
| **3 (201; 1)** | positive | positive | positive | 12 | lung | N/A |
| **4 (202; 1)** | positive | positive | positive | 63 | gall bladder | N/A |
| **5 (202; 1)** | positive | positive | positive | 42 | gall bladder | N/A |
| **6 (203; 1)** | positive | positive | positive | 40 | lung | positive |
| **7 (203; 1)** | positive | positive | positive | 96 | lung | positive |
| **8 (204; 1)** | positive | positive | positive | 110 | lung | positive |
| **9 (204; 1)** | positive | positive | positive | 21 | lung | positive |
| **10 (204; 1)** | positive | positive | positive | 15 | lung | positive |
| **11 (204; 1)** | positive | positive | positive | 7.5 | lung | positive |
| **12 (204; 2)** | positive | positive | positive | 77 | lung | positive |
| **13 (204; 2)** | positive | positive | positive | 54 | lung | positive |
| **14 (204; 3)** | positive | positive | positive | 44 | lung | positive |
| **15 (205; 1)** | positive | positive | negative | 20.25 | lung | N/A |
| **16 (205; 1)** | positive | positive | positive | 36 | lung | N/A |
| **17 (205; 1)** | positive | negative | positive | 45 | lung | N/A |
| **18 (205; 1)** | negative | negative | negative | 12 | lung | N/A |
| **19 (205; 1)** | positive | positive | negative | 20 | lung | N/A |
| **20 (205; 2)** | positive | positive | positive | 70 | lung | N/A |
| **21 (205; 2)** | positive | positive | positive | 18 | lung | N/A |
| **22 (205; 2)** | positive | positive | positive | 21 | lung | N/A |
| **23 (205; 3)** | positive | positive | positive | 21 | lung | N/A |
| **24 (205; 3)** | positive | positive | positive | 26.25 | lung | N/A |
| **25 (206; 1)** | positive | positive | positive | 24.5 | gall bladder | N/A |
| **26 (206; 2)** | positive | positive | positive | 10 | liver | N/A |
| **27 (207; 1)** | positive | positive | positive | 4 | gall bladder | negative |
| **28 (208; 1)** | positive | positive | positive | 24 | gall bladder | positive |
| **29 (209; 1)** | positive | positive | positive | 98 | liver | positive |
| **30 (209; 1)** | positive | positive | positive | 84 | liver | positive |
| **31 (210; 1)** | positive | positive | negative | 6.25 | gall bladder | positive |
| **32 (211; 1)** | positive | positive | positive | 210 | liver | positive |
| **33 (211; 2)** | positive | positive | positive | 189 | liver | positive |
| **34 (211; 2)** | positive | positive | positive | 42 | liver | positive |
| **35 (211; 2)** | positive | positive | positive | 35 | liver | positive |
| **36 (211; 2)** | positive | positive | positive | 12 | liver | positive |
| **37 (212; 1)** | positive | positive | negative | 24 | vena cava | positive |
| **38 (212; 2)** | positive | positive | negative | 140 | duodenum | positive |
| **39 (212; 2)** | positive | positive | negative | 77 | duodenum | positive |
| **40 (212; 2)** | positive | positive | negative | 48 | duodenum | positive |
| **41 (212; 2)** | negative | negative | negative | 10 | duodenum | positive |
