# Supplementary material for: Immunohistological detection of small particles of Echinococcus multilocularis and Echinococcus granulosus in lymph nodes is associated with enlarged lymph nodes in alveolar and cystic echinococcosis
Source: PLoS Negl Trop Dis. 2020 Dec 28;14(12):e0008921. doi: 10.1371/journal.pntd.0008921 (PMC7769273; doi:10.1371/journal.pntd.0008921)
Supplement: S5 Table — (DOCX) [file pntd.0008921.s005.docx]

**S5 Table. Data of control lymph nodes (n=74) of 8 uninfected patients**

| **lymph node** | **cohort** | **area (mm²)** | **localization** |
| --- | --- | --- | --- |
| **lymph node 1  (patient 301; slide 1)** | abdomen | 30 | gall bladder |
| **2 (302; 1)** | abdomen | 24 | gall bladder |
| **3 (303; 1)** | abdomen | 35 | gall bladder |
| **4 (304; 1)** | abdomen | 8 | colon |
| **5 (304; 1)** | abdomen | 7,5 | colon |
| **6 (304; 1)** | abdomen | 1 | colon |
| **7 (304; 1)** | abdomen | 28 | colon |
| **8 (304; 1)** | abdomen | 12 | colon |
| **9 (304; 2)** | abdomen | 9 | colon |
| **10 (304; 2)** | abdomen | 20 | colon |
| **11 (304; 2)** | abdomen | 30 | colon |
| **12 (304; 2)** | abdomen | 35 | colon |
| **13 (304; 2)** | abdomen | 1 | colon |
| **14 (304; 3)** | abdomen | 35 | colon |
| **15 (304; 3)** | abdomen | 15 | colon |
| **16 (304; 3)** | abdomen | 12 | colon |
| **17 (304; 3)** | abdomen | 0,5 | colon |
| **18 (304; 3)** | abdomen | 10 | colon |
| **19 (304; 3)** | abdomen | 7,5 | colon |
| **20 (304; 4)** | abdomen | 28 | colon |
| **21 (304; 4)** | abdomen | 45 | colon |
| **22 (304; 5)** | abdomen | 4 | colon |
| **23 (305; 1)** | abdomen | 30 | rectum |
| **24 (305; 1)** | abdomen | 25 | rectum |
| **25 (305; 1)** | abdomen | 7 | rectum |
| **26 (305; 2)** | abdomen | 48 | rectum |
| **27 (305; 2)** | abdomen | 25 | rectum |
| **28 (305; 2)** | abdomen | 5 | rectum |
| **29 (305; 3)** | abdomen | 22,5 | rectum |
| **30 (305; 4)** | abdomen | 4 | rectum |
| **31 (305; 5)** | abdomen | 1 | rectum |
| **32 (306; 1)** | thorax | 5,25 | lung |
| **33 (306; 2)** | thorax | 36 | lung |
| **34 (306; 2)** | thorax | 31,5 | lung |
| **35 (306; 3)** | thorax | 35 | lung |
| **36 (306; 3)** | thorax | 2 | lung |
| **37 (306; 4)** | thorax | 6 | lung |
| **38 (306; 4)** | thorax | 9 | lung |
| **39 (306; 4)** | thorax | 27 | lung |
| **40 (306; 5)** | thorax | 8 | lung |
| **41 (306; 6)** | thorax | 45 | lung |
| **42 (306; 6)** | thorax | 50 | lung |
| **43 (307; 1)** | thorax | 12 | lung |
| **44 (307; 2)** | thorax | 36 | lung |
| **45 (307; 2)** | thorax | 3 | lung |
| **46 (307; 3)** | thorax | 21 | lung |
| **47 (307; 4)** | thorax | 24 | lung |
| **48 (307; 5)** | thorax | 6 | lung |
| **49 (307; 5)** | thorax | 3 | lung |
| **50 (307; 5)** | thorax | 9 | lung |
| **51 (307; 5)** | thorax | 6 | lung |
| **52 (307; 5)** | thorax | 32 | lung |
| **53 (307; 5)** | thorax | 35 | lung |
| **54 (307; 6)** | thorax | 24 | lung |
| **55 (307; 6)** | thorax | 16 | lung |
| **56 (307; 6)** | thorax | 20 | lung |
| **57 (307; 6)** | thorax | 88 | lung |
| **58 (307; 6)** | thorax | 15 | lung |
| **59 (307; 7)** | thorax | 1 | lung |
| **60 (307; 8)** | thorax | 40 | lung |
| **61 (307; 8)** | thorax | 24 | lung |
| **62 (307; 8)** | thorax | 3 | lung |
| **63 (307; 8)** | thorax | 36 | lung |
| **64 (307; 8)** | thorax | 110 | lung |
| **65 (308; 1)** | thorax | 24 | lung |
| **66 (308; 2)** | thorax | 110 | lung |
| **67 (308; 2)** | thorax | 45 | lung |
| **68 (308; 3)** | thorax | 24 | lung |
| **69 (308; 3)** | thorax | 20 | lung |
| **70 (308; 4)** | thorax | 60 | lung |
| **71 (308; 5)** | thorax | 50 | lung |
| **72 (308; 5)** | thorax | 8 | lung |
| **73 (308; 6)** | thorax | 6 | lung |
