# Supplementary material for: Immunohistological detection of small particles of Echinococcus multilocularis and Echinococcus granulosus in lymph nodes is associated with enlarged lymph nodes in alveolar and cystic echinococcosis
Source: PLoS Negl Trop Dis. 2020 Dec 28;14(12):e0008921. doi: 10.1371/journal.pntd.0008921 (PMC7769273; doi:10.1371/journal.pntd.0008921)
Supplement: S6 Table — (DOCX) [file pntd.0008921.s006.docx]

**S6 Table. Data of lymph nodes per patient**

| **patient** | **group** | **mAb EmG3** | **area (mm²)** | | | | |
| --- | --- | --- | --- | --- | --- | --- | --- |
|  |  |  | **mean** | **standard deviation** | **median** | **max** | **min** |
| **101** | AE | positive | 276.00 | N/A | 276.00 | 276.00 | 276.00 |
| **102** | AE | positive | 53.50 | 31.91 | 56.00 | 82.50 | 4.00 |
| **103** | AE | positive | 20.13 | 8.66 | 20.13 | 26.25 | 14.00 |
| **104** | AE | positive | 56.33 | 81.16 | 12.00 | 150.00 | 7.00 |
| **105** | AE | positive | 133.33 | 80.21 | 140.00 | 210.00 | 50.00 |
| **106** | AE | positive | 109.33 | 62.14 | 128.00 | 160.00 | 40.00 |
| **107** | AE | positive | 35.00 | 44.74 | 4.00 | 84.00 | 1.00 |
| **108** | AE | positive | 55.17 | 64.11 | 36.00 | 208.00 | 4.00 |
| **109** | AE | positive | 64.65 | 68.44 | 37.25 | 200.00 | 12.00 |
| **110** | AE | positive | 40.00 | N/A | 40.00 | 40.00 | 40.00 |
| **111** | AE | negative | 27.67 | 21.50 | 28.00 | 49.00 | 6.00 |
| **112** | AE | negative | 18.00 | N/A | 18.00 | 18.00 | 18.00 |
| **113** | AE | positive | 44.00 | 28.28 | 44.00 | 64.00 | 24.00 |
| **114** | AE | positive | 15.75 | 19.96 | 7.50 | 45.00 | 3.00 |
| **115** | AE | positive | 18.05 | 14.62 | 12.00 | 35.00 | 4.50 |
| **116** | AE | positive | 51.62 | 42.48 | 50.00 | 160.00 | 4.00 |
| **117** | AE | positive | 55.50 | 40.31 | 55.50 | 84.00 | 27.00 |
| **118** | AE | negative | 89.33 | 76.51 | 91.00 | 165.00 | 12.00 |
| **119** | AE | positive | 20.50 | 27.58 | 20.50 | 40.00 | 1.00 |
| **120** | AE | positive | 162.00 | 103.55 | 194.00 | 240.00 | 20.00 |
| **121** | AE | positive | 93.20 | 149.54 | 33.00 | 360.00 | 8.00 |
| **122** | AE | negative | 36.00 | N/A | 36.00 | 36.00 | 36.00 |
| **123** | AE | positive | 185.20 | 169.57 | 84.00 | 378.00 | 24.00 |
| **124** | AE | positive | 15.00 | N/A | 15.00 | 15.00 | 15.00 |
| **125** | AE | positive | 32.75 | 44.19 | 32.75 | 64.00 | 1.50 |
| **201** | CE | positive | 9.50 | 4.33 | 12.00 | 12.00 | 4.50 |
| **202** | CE | positive | 52.50 | 14.85 | 52.50 | 63.00 | 42.00 |
| **203** | CE | positive | 68.00 | 39.60 | 68.00 | 96.00 | 40.00 |
| **204** | CE | positive | 46.93 | 36.94 | 44.00 | 110.00 | 7.50 |
| **205** | CE | positive | 28.95 | 17.29 | 21.00 | 70.00 | 12.00 |
| **206** | CE | positive | 17.25 | 10.25 | 17.25 | 24.50 | 10.00 |
| **207** | CE | positive | 4.00 | N/A | 4.00 | 4.00 | 4.00 |
| **208** | CE | positive | 24.00 | N/A | 24.00 | 24.00 | 24.00 |
| **209** | CE | positive | 91.00 | 9.90 | 91.00 | 98.00 | 84.00 |
| **210** | CE | positive | 6.25 | N/A | 6.25 | 6.25 | 6.25 |
| **211** | CE | positive | 97.60 | 93.97 | 42.00 | 210.00 | 12.00 |
| **212** | CE | positive | 59.80 | 51.55 | 48.00 | 140.00 | 10.00 |
| **301** | control | N/A | 30.00 | N/A | 30.00 | 30.00 | 30.00 |
| **302** | control | N/A | 24.00 | N/A | 24.00 | 24.00 | 24.00 |
| **303** | control | N/A | 35.00 | N/A | 35.00 | 35.00 | 35.00 |
| **304** | control | N/A | 16.24 | 13.38 | 12.00 | 45.00 | 0.50 |
| **305** | control | N/A | 18.61 | 15.55 | 22.50 | 48.00 | 1.00 |
| **306** | control | N/A | 23.16 | 17.55 | 27.00 | 50.00 | 2.00 |
| **307** | control | N/A | 25.64 | 26.90 | 20.50 | 110.00 | 1.00 |
| **308** | control | N/A | 38.56 | 32.59 | 24.00 | 110.00 | 6.00 |

*N/A: not available; ^1^positive if one positive lymph node*
