# Supplementary figures and images for: Immunohistological detection of small particles of Echinococcus multilocularis and Echinococcus granulosus in lymph nodes is associated with enlarged lymph nodes in alveolar and cystic echinococcosis
Source: PLoS Negl Trop Dis. 2020 Dec 28;14(12):e0008921. doi: 10.1371/journal.pntd.0008921 (PMC7769273; doi:10.1371/journal.pntd.0008921)

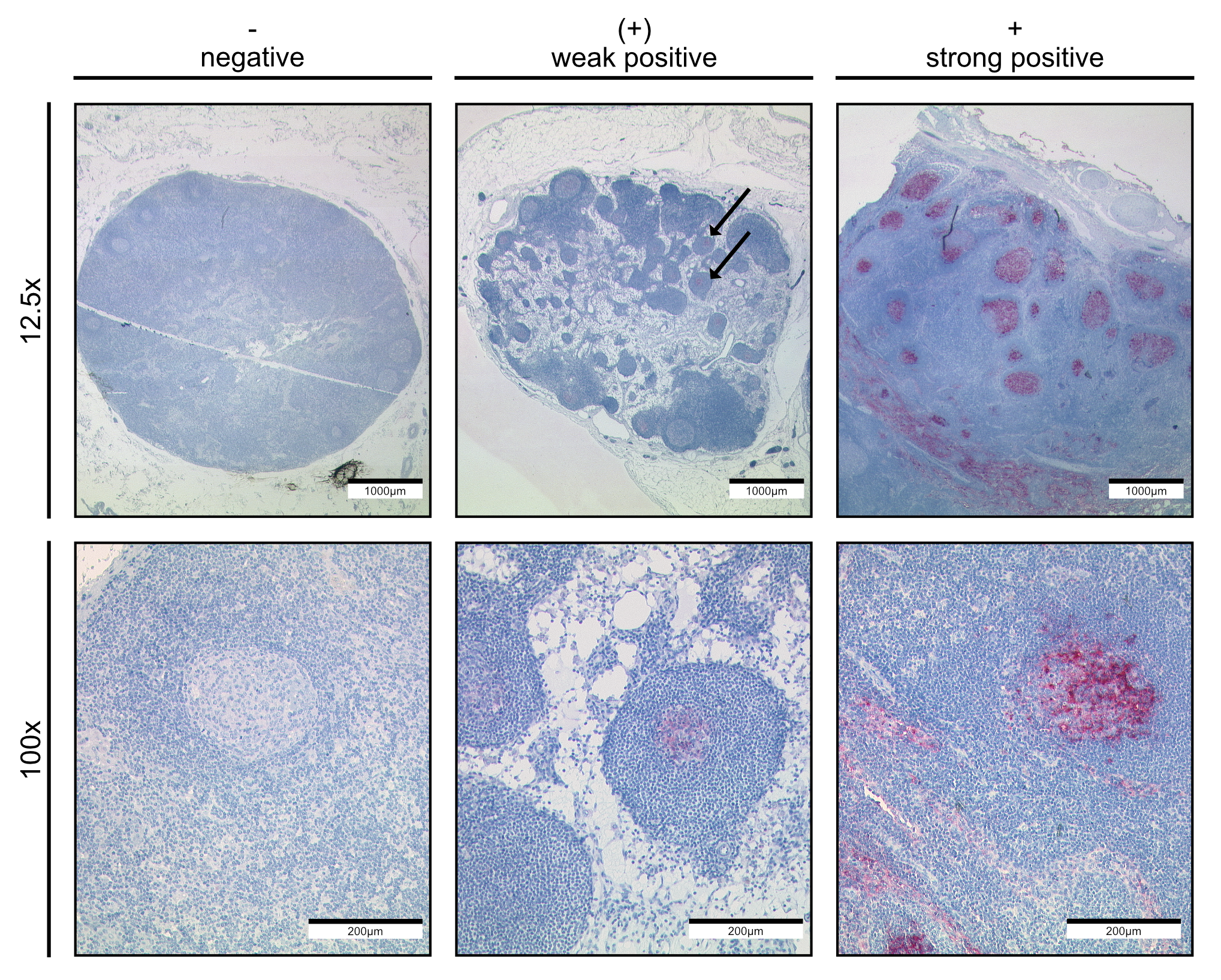

Supplement: S1 Fig — (-) no staining in both magnifications. ((+)) weak positive staining in 100x magnification; (+) strong positive staining in both magnifications. (TIF) [file pntd.0008921.s007.tif]

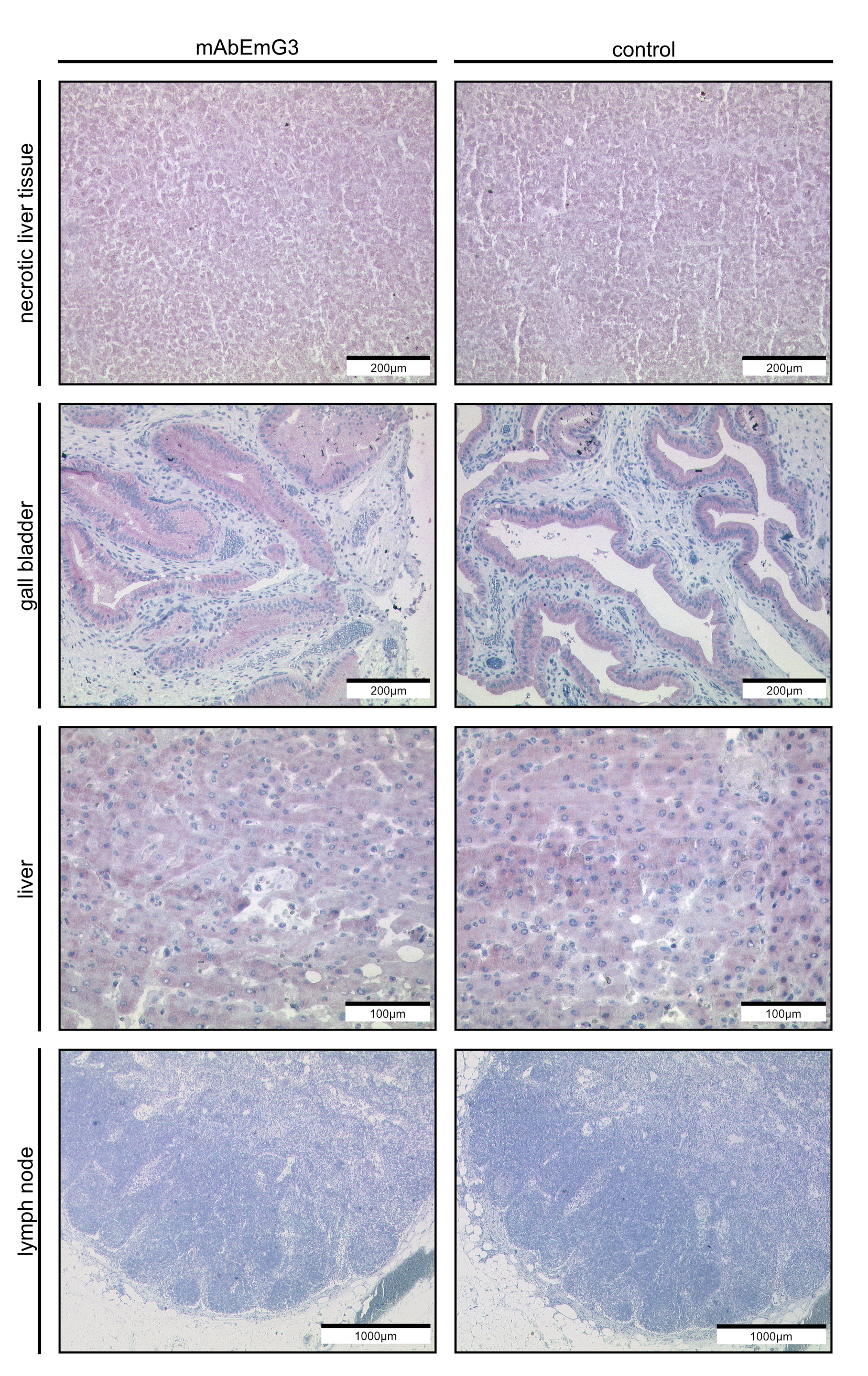

Supplement: S2 Fig — (left) mAb EmG3-IHC shows no specific staining. (right) Control staining without primary antibody. Only weak background staining is seen in hepatocytes and gall bladder epithelia. (TIF) [file pntd.0008921.s008.tif]

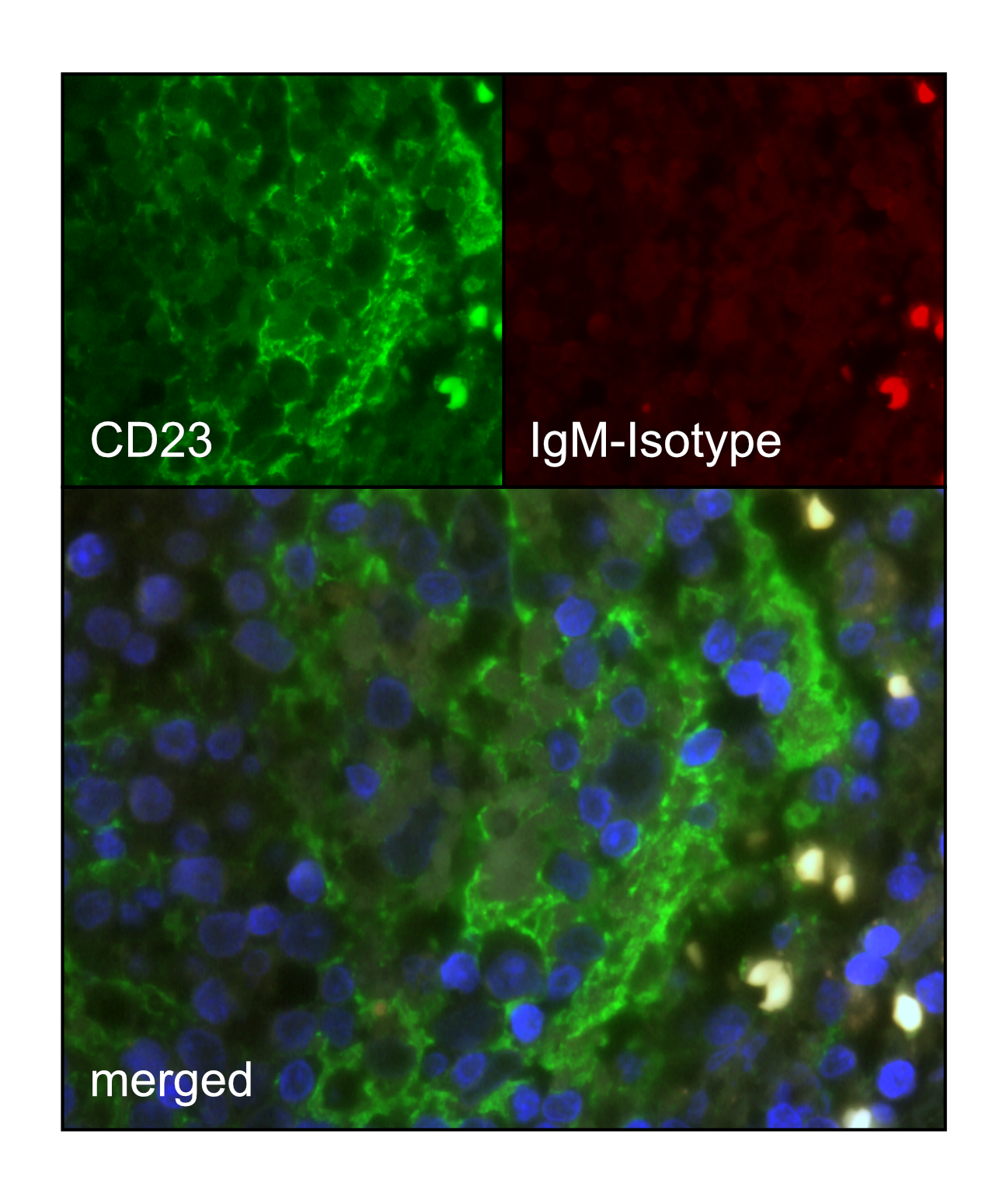

Supplement: S3 Fig — Double-immunofluorescence staining with CD23 (green) and IgM-isotype control (red) of a CE lymph node. No staining in isotype control. (TIF) [file pntd.0008921.s009.tif]
