# Supplementary material for: Immunohistological detection of small particles of Echinococcus multilocularis and Echinococcus granulosus in lymph nodes is associated with enlarged lymph nodes in alveolar and cystic echinococcosis
Source: PLoS Negl Trop Dis. 2020 Dec 28;14(12):e0008921. doi: 10.1371/journal.pntd.0008921 (PMC7769273; doi:10.1371/journal.pntd.0008921)
Supplement: Attachment — Submitted filename: Cover_Letter_lymph_nodes.docx [file pntd.0008921.s010.docx]

PLOS Neglected Tropical Diseases

**Immunohistological Detection of Small Particles of Echinococcus multilocularis and Echinococcus granulosus in Lymph Nodes is Associated with Enlarged Lymph Nodes in Alveolar and Cystic Echinococcosis**

September 11, 2020

**INSTITUT FÜR PATHOLOGIE**

**Ärztlicher Direktor**

Prof. Dr. med. Peter Möller

Albert-Einstein-Allee 23

89070 Ulm

**Sekretariat**

elke.carter@uniklinik-ulm.de

T: 0731 500-56321

F: 0731 500-56384

**UNIVERSITÄTSKLINIKUM ULM** | Institut für Pathologie | 89070 Ulm

Dear Editors, dear Reviewers,

First, we would like to thank the editor and the reviewers for the expert reading and their comments which certainly helped to improve the quality of our manuscript. In particular, we would like to answer to the questions as follows:

*Editor:*

1) **Please provide information on the distribution of samples per patient (mean, SD, median, interquartile range, maximum, and the minimum number of samples per patient). These data might come in Table 2 or a supplementary table.**

Thank you for this recommendation. We provided these data in Table S6. In Table S3-5 we changed the numbering of the patient in order that the numbering is consistent.

2) **Most analyses are performed at the lymph node level (e.g., comparing sizes), but this approach has an inherent statistical problem since lymph nodes are clustered within individuals. In this situation, the characteristics of lymph nodes might be more similar within individuals than between individuals. A more appropriate statistical approach taking care of this random effect should have been considered using, for instance, a mixed-effects ANOVA for comparing means between groups.**

This is a correct observation. Such analysis would be an improvement for our study. We tried together with the department of statistic to solve this problem. Unfortunately, our cohort is limited and analysis with ANOVA model in SAS provided no valid results (see appendix). We discussed this problem with the statistic department of our University and together with these experts we decided that we cannot provide further solid statistical analysis for the lymph nodes results as requested. However, from a biological point of view, a lymph node is a dynamic structure and the size is a function of the draining area which might be inflamed or not. Therefore, function is reflected by the size of the lymph node. Therefore, we have pointed out this limitation in our manuscript (line 389): “Due to a limited number of lymph nodes the statistical analysis is restricted. Therefore, we cannot provide valid data of the influence of individual differences of lymph node size.”

3) **Please check Table 3 and confirm that among AE (affected with spems; n=63), the numbers of strong positive and weak negative are correct (they sum up 50). If yes, please provide a legend for the other 13 samples that were not strong or weak positive.**

The reviewer is right, this is a mistake. Thank you for this observation. The 13 samples are part of the “weak positive” group. We corrected Table 3 accordingly.

*Reviewer 1:*

Thank you for this positive feedback.

1) **The majority of the parts that require correction consist of simple typing error. Please check for typing errors in the entire manuscript.**

Thank you for the exact reading! We have corrected the mentioned typos. Further, we checked the MS again for errors.
The journal abbreviations were inserted; in line 455 we do not find an error (the abbreviation of *Parasitology* is to our knowledge *Parasitology (<https://www.ncbi.nlm.nih.gov/nlmcatalog/401121>*).

*Reviewer 2:*
Many thanks for this feedback. This helped to improve our manuscript.

1) **Ref to the significance of the study, the authors failed to make a compelling case for the clinical and diagnostic implications and/or advantages of using mAb-based diagnosis. No comparisons have been made with FFPE-based molecular techniques which seem to be less expensive and more reliable. As the authors pointed out IHC with the mAbs could be an adjunct for the final histological diagnosis, in addition to the routine H&E staining that poses high diagnostic value. This is particularly true for CE.**
   The reviewer discovered an error made by us. We missed the comparison of immunohistochemistry and molecular methods as PCR. We are grateful for this hint and added a short passage to describe the advantages of immunohistochemistry (see line 315)

The corrected text is as follows: “In addition to conventional histology, PCR is assumed to be the gold standard in diagnosis of echinococcosis. Reinehr et al. showed that combined immunohistochemical staining with mAb Em2G11 and mAb EmG3 is as sensitive and specific as PCR. Compared to PCR, immunostaining is faster and less expensive. Moreover, immunohistochemistry is available in many laboratories around the world, which have not the possibility to perform PCR [1]

CE is a worldwide burden, with endemic areas also in rural regions and locations with no possibility for PCR techniques [2]. Patients in these areas could benefit from the immunohistological diagnostic process using the mAb EmG3.

Furthermore, in our daily routine diagnostics we have observed single cases of echinococcosis with detectable spems or spegs, which were negative by PCR.” (Manuscript submitted)

2) **Did you genotype the parasites? If yes, did you find any evidence of variation within different species/genotype of E. granulosus sensu lato?**
   This is an interesting question and warrants further analysis which yet has not be done. This question lies outside the focus of this study.
3) **No comparisons have been made with the initial clinical diagnosis of the patients. Were there any patients with an initial diagnosis of CE that is proved to be AE by mAb-IHC, and vice versa? This is specially important in the countries like China, Turkey and Iran where both CE and AE are co-endemic.**
   This is an interesting question with high relevance not only in the endemic areas. As you have noticed mAb EmG3 does not allow a distinction between AE and CE. Our entire cohort has been stained with the mAb Em2G11 and the results have been published. Indeed, in this study we showed that some cases had to be reclassified after immunohistochemistry with mAb Em2G11 [3]
   With the new mAb EmG3 we were able to define 8 cases as CE, which were suspicious for echinococcosis, not otherwise specified.
4) **The authors use WHO-PNM classification for AE, however no detailed data were provided for WHO-IWGE classification of CE lesions.**

That is a good point. Unfortunately, we are not able to classify the CE lymph node cohort according to the WHO-IGWE. Most of the CE cases are older than 10 years. Three cases are from before 2000. The WHO-IGWE classification was established in 2003 (<https://pubmed.ncbi.nlm.nih.gov/12606104/> ). Furthermore, we do not have the ultrasonic images. To our regret, we cannot provide this classification of the CE cases.

5) **About 20% of AE samples originated from the lymph nodes. Does it mean 25% of AE patients have AE cystic lesions in their lymph nodes? The same is more or less true about CE, i.e. 10.7% of samples and 15.6% of CE patients. These are odd figures. Were they primary or metastatic lesions?**

We are grateful for this hint. Obviously, we are failed to clearly present our data in Table 1. The numbers of lymph node samples refer to the absolute number of samples of AE or CE infected patient; this table does not include information regarding the IHC results. To clarify this: Only three AE and three CE lymph node samples showed histological detectable fragments of the laminated layer as detected by conventional histology (HE and PAS).

We included these data in the result section line 218. We apologize for this error.

6) **Figures 1 and 3 resolutions are not adequate to support findings of the study.**
   We are sorry to hear this. The resolution of our original files was high (Figure 1: 2480 x 1816, 300dpi; Figure 3: 2363 x 3336, 300dpi). We think an error occurred in uploading process. To fix this, we will upload new files (PDF) of the figures.
7) **line 215: "IHC with mAb EmG3 revealed positive staining of fragments of the laminated layer and hence diagnosis of CE was confirmed." As EmG3 is genus-specific and reacts with both AE and CE, how did you reach a CE diagnosis rather than AE?**
   We thank the reviewer for this comment. Our wording was not precise and has led to this misunderstanding. We now specified this paragraph as following (line 222):

“We included 8 cases of echinococcosis which were previously classified as suspicious for echinococcosis. All 8 cases did not show an intact laminated layer or protoscoleces in the HE and PAS staining. There was no immunoreactivity in the staining with mAb Em2G11, therefore diagnosis of AE seemed to be unlikely. IHC with mAb EmG3 revealed positive staining of fragments of the laminated layer; in combination with the negative mAb Em2G11 staining the diagnosis of CE was confirmed (diagnostic algorithm introduced in Reinehr et al. [1]).”

8) **line 232: "In 39 of 41 (95.1%) lymph nodes of CE patients we detected spegs." Please provide the number of positive patients as well (? out of 12 patients?).**
   Thank you for this comment. We now provide the data (CE:12 of 12; AE: 21 of 25) and changed the manuscript accordingly.
9) **The authors found spegs and traces of laminated layer in 95% of lymph nodes of CE patients with lymph node involvements. Were there any patients with organ involvements other than lymph nodes, positive for spegs in the lymph nodes? Same comment for spems.**
   We thank the reviewer for this interesting question. As described in the text (line 209) we detected spegs and spems in the main lesions, adjacent to the main lesions in blood vessels and in draining lymph nodes. We did not see spegs or spems in other regions or organs. However, this statement is of limited informative value. We did not have the opportunity to analyze further tissues of our patients. This was a retrospective study of an existing cohort and was limited to the available material. No further biopsies of other organs are available. Of course, it is of high interest to analyze further organs. To answer this question a prospect study with multiple biopsies is needed. What we know by now is that spems do not contain DNA, therefore spems are unlikely to present infectious material (unpublished manuscript).
10) **No statistical analysis and P-values were provided in Table 3.**
    Thank you for this hint. We insert the p-values in Table 3.
11) **The authors could discuss the value of SEM images in this study and recommendations for future studies.**
    That is an interesting consideration. We are currently performing analyses by electron microscopy. Data are still too preliminary and therefore are not included in the manuscript.
12) **Minor comments: Typing errors**
    Thank you for the exact reading. We corrected the mistakes.

*Reviewer 3:*

Thank you for your positive feedback and your observation.

1) **Author Summary. Line 30. echinococcosis with lower case.**
   Thank you for this observation. We corrected the error.
2) **Scientific names should be abbreviated after its first appearance on the text. Revise the entire manuscript. Example, line 34: change "Echinococcus granulosus s.l." to "E. granulosus s.l.".**
   Thank you for this feedback. We checked the manuscript and changed the scientific names.
3) **Lines 90 and 91. "Cystic formations". Revise the use of the word "cyst" for alveolar echinococcosis. "Cyst" should never be used to designate the central necrotic cavity often developed in the AE lesions. “Pseudocyst” should be used for this cavity. See: International consensus on terminology to be used in the field of echinococcoses. Vuitton et al. 2020 (Parasite 27, 41).**
   Thank you for this comment. We changed the section and avoided the use of this term (see line 92).
4) **Line 113. If the authors underline the first letter to show the acronym of "spegs", please do the same in line 109 for "spems".**
   Thank you for this observation. We changed it in the introduction and the abstract.
5) **Line 236. protoscoleces in lower case.**
   Done.

**Further changes:**

- In line 152 we specified the used concertation of mAb Em2G11 and mAb EmG3.
- We added in the ethics statement a part that outline the anonymization of patient data.

We are very grateful for the editor`s and reviewers` advice which helped us a lot to significantly improve our manuscript. We hope that our reworked manuscript is now deemed suitable for publication in PLOS Neglected Tropical Diseases. On behalf of the authors,

Prof. Dr. med. Thomas FE Barth

**References**

1. Reinehr M, Micheloud C, Grimm F, Kronenberg PA, Grimm J, Beck A, et al. Pathology of Echinococcosis: A Morphologic and Immunohistochemical Study on 138 Specimens With Focus on the Differential Diagnosis Between Cystic and Alveolar Echinococcosis. Am J Surg Pathol. 2019. doi: 10.1097/PAS.0000000000001374 [doi].

2. Agudelo Higuita NI, Brunetti E, McCloskey C. Cystic Echinococcosis. J Clin Microbiol. 2016;54: 518-523. doi: 10.1128/JCM.02420-15 [doi].

3. Barth TF, Herrmann TS, Tappe D, Stark L, Gruner B, Buttenschoen K, et al. Sensitive and specific immunohistochemical diagnosis of human alveolar echinococcosis with the monoclonal antibody Em2G11. PLoS Negl Trop Dis. 2012;6: e1877. doi: 10.1371/journal.pntd.0001877 [doi].
