# Supplementary material for: Immunohistological detection of small particles of Echinococcus multilocularis and Echinococcus granulosus in lymph nodes is associated with enlarged lymph nodes in alveolar and cystic echinococcosis
Source: PLoS Negl Trop Dis. 2020 Dec 28;14(12):e0008921. doi: 10.1371/journal.pntd.0008921 (PMC7769273; doi:10.1371/journal.pntd.0008921)
Supplement: Attachment — Submitted filename: Response_letter_rev2.docx [file pntd.0008921.s011.docx]

PLOS Neglected Tropical Diseases

**Immunohistological Detection of Small Particles of Echinococcus multilocularis and Echinococcus granulosus in Lymph Nodes is Associated with Enlarged Lymph Nodes in Alveolar and Cystic Echinococcosis**

September 30, 2020

**INSTITUT FÜR PATHOLOGIE**

**Ärztlicher Direktor**

Prof. Dr. med. Peter Möller

Albert-Einstein-Allee 23

89070 Ulm

**Sekretariat**

elke.carter@uniklinik-ulm.de

T: 0731 500-56321

F: 0731 500-56384

**UNIVERSITÄTSKLINIKUM ULM** | Institut für Pathologie | 89070 Ulm

Dear Editors, dear Reviewers,

we are pleased to hear this positive feedback.

We handled the two open questions and adjusted our manuscript:

1. **Editor, ref to the comment #2: I suggest changing the phrase included (“Due to a limited number of lymph nodes the statistical analysis is restricted. Therefore, we cannot provide valid data of the influence of individual differences of lymph node size.”) by the following ("Since more than one lymph node was sampled within individuals, the characteristics of lymph nodes might be more similar within individuals than between individuals, and a proper statistical approach would have to consider this effect. However, due to a limited number of lymph nodes, the statistical analysis is restricted, and we could not provide valid data of the influence of individual differences on lymph node size.”)**
   Thank you for this suggestion. This formulation makes the limitation and the reasons for it clear. We have integrated the sentence accordingly (line 381)
2. **Reviewer #2, ref to the comment #4: please add statements in the manuscript regarding this limitation.**
   Thank you for this observation. We are aware of this limitation. We insert the following sentence: “Due to limitation of clinical data (e.g. missing ultrasound images) we could not provide the WHO Informal Working Group ultrasound classification for the CE cases.” (line 176)
3. **Thank you for including your ethics statement: "Approval for this study was obtained from the local Ethics Committee (vote for usage of archived human material 03/2014) and was in compliance with the ethical principles of the WMA Declaration of Helsinki (see: Dtsch Arztebl. 2003: 1632); patient data were anonymized accordingly." Please include the full name(s) of the Institutional Review Board(s) or Ethics Committee(s).**

We include the full name: “Ethics Committee of the university of Ulm”

We hope, we handled these issues according to your convenience.

On behalf of the authors,

Prof. Dr. med. Thomas FE Barth
